# Government Experts Favor Extermination of Rats

**Published:** 1918-08

**Authors:** 


### Government Experts Favor Extermination of Rats Also as a War Measure for Saving Food.

Government experts are urging that the rat be exterminated as a war measure for saving food. The waste each year due to the rodent is estimated at $200,000,000. A full-grown rat consumes more food than a baby. In addition, the animal is a menace to health.

The terrible scourge of the bubonic plague in Europe and Asia was spread by rats and their parasites. When the plague was carried by the animals in ships to our Pacific coast, a campaign for their extermination was conducted in the seaports at much cost. The disease was thus stamped out as it had been in the Orient, by the pound of cure instead of the ounce of prevention.

---

THE PROFESSOR OF LOGIC *(to himself)*:—I laid my hat somewhere in this room. Nobody has come in since I've been here. I can't see it anywhere; therefore *(putting his hand beneath him)* I have sat on it. Another proof of the irrestible power of logic.

---

Q:—What is the first thing that you would say to a Twilight Baby?

A:—Does your mother know that you're out?—T. H.

